# The upper limit for TSH during pregnancy: why we should stop using fixed limits of 2.5 or 3.0 mU/l

**DOI:** 10.1186/s13044-018-0048-7

**Published:** 2018-05-21

**Authors:** Tim I. M. Korevaar

**Affiliations:** Department of Internal Medicine, Erasmus University Medical Center, Rotterdam Academic Center for Thyroid Disease, Erasmus University Medical Center, Wytemaweg 80, 3015 CN Rotterdam, Rotterdam, The Netherlands

**Keywords:** Thyroid, Pregnancy, FT4, Reference range

## Abstract

Physiological changes necessitate the use of pregnancy-specific reference ranges for TSH and FT4 to diagnose thyroid dysfunction during pregnancy. Although many centers use fixed upper limits for TSH of 2.5 or 3.0 mU/L, this comment describeds new data which indicate that such cut-offs are too low and may lead to overdiagnosis or even overtreatment. The new guidelines of the American Thyroid Association have considerably changed recommendations regarding thyroid function reference ranges in pregnancy accordingly. Also a stepwise approach to interpreting these guidelines is discussed as well as the relevant role of FT4 in diagnosis.

## Background

Thyroid physiology changes during pregnancy and this necessitates the use of pregnancy-specific reference ranges for TSH and FT4 in order to adequately diagnose gestational thyroid disease [1]. Currently, many centers use a reference range for TSH with an upper limit of 2.5 mU/l in the first trimester and 3.0 mU/l in the second or third trimester to diagnose subclinical and overt hypothyroidism. This is based on outdated international guidelines from the American Thyroid Association (2011), the Endocrine Society (2012) and the European Thyroid Association (2014) [2–4]. Although each of these guidelines recommend to calculate lab-specific reference ranges for TSH and FT4, many centers do not have such reference ranges available. Instead, most centers adhere to the former second recommendation which is that, in the absence of lab-specific reference ranges, fixed upper limits for TSH (2.5 mU/l in the first and 3.0 mU/l in the second and third trimester) can be used. However, recent studies have indicated that these cut-offs are too low and may lead to overdiagnosis and unnecessary treatment, or even overtreatment. Based on some important findings discussed below, the 2017 American Thyroid Association guidelines have updated the recommendations on the upper limit for TSH during pregnancy.

## Main text

Various studies have demonstrated that with the use of fixed TSH upper limits, 8–28% of pregnant women have a TSH concentration that is considered too high [5, 6]. These numbers are much larger than the roughly 3–4% that would have a too high TSH if population-based reference ranges would be used to define the upper limits for TSH. Medicalization of a group of women as large as 8–28% is unwarranted, unsustainable and likely to cause more harm than benefit. Further data indicate that the upper limit for TSH should be higher. By summarizing 14 studies that calculated population-based pregnancy-specific reference ranges for TSH and/or FT4, our group was able to show that in more than 90% of all studies, the upper limit for TSH was above 2.5 or 3.0 mU/l [7]. Moreover, the few studies performed in a population that was proven to be iodine sufficient report an upper limit for TSH of 4.04 and 4.34 mU/l [7], however, the effects of population iodine status on reference range values remains to be studied. Interestingly, a large randomized controlled trial that screened approximately 100.00 pregnant women for subclinical hypothyroidism and hypothyroxinemia using the fixed TSH cut-offs [8] had to amend its protocols because the TSH upper limit turned out to be 4.0 mU/l after roughly 15.000 women were screened.

The 2017 ATA guidelines [9] now recommend the following:

1. Calculate pregnancy-specific and lab-specific references ranges for TSH and FT4
2. If 1 is not possible, adopt a reference range from the literature that is derived using a similar assay and preferably also in a population with similar characteristics (i.e. ethnicity, BMI, iodine status)
3. If 1 and 2 are not possible, deduct 0.5 mU/l from the non-pregnancy reference range (which in most centers would results in a cut-off of roughly 4.0 mU/l)

My interpretation of these recommendations is probably more strict than that of most endocrinologists or gynecologists. Lab-specific reference ranges better identify women with gestational thyroid dysfunction than reference ranges defined by another methodology [7, 10]. Calculating lab-specific references ranges is not difficult and every hospital in which prenatal care is provided would be able to perform a good study at very low costs (i.e. less than a few thousand euro/GBP), particularly when collaborating with the clinical chemistry department. Adequate reference ranges can be obtained by selecting at least 400 pregnant women with a singleton pregnancy, free of pre-existing thyroid disease, that do not use thyroid interfering medication, that did not undergo IVF treatment and are TPOAb negative [7]. Therefore, I believe that if a center does not have lab-specific reference ranges readily available, physicians should not automatically move to step 2 or 3 of the guideline recommendations, but try to obtain lab-specific reference ranges. Calculating such reference ranges will instantly improve the quality of clinically diagnosing thyroid dysfunction in pregnancy. When specific expertise is missing, groups involved in the field of thyroid and pregnancy (including our group) would be more than willing to share their experience.

Although it seems clear that fixed upper TSH limits of 2.5 mU/l or 3.0 mU/l can no longer be considered adequate, the new ATA guidelines seem to make one exception. A new recommendation indicates that levothyroxine treatment can be considered for a TSH above the reference range in TPOAb negative women, while for TPOAb positive women treatment can be considered from a TSH above 2.5 mU/L [9]. This is based on data from observational studies showing that there is a higher risk of miscarriage and premature delivery in TPOAb positive women with high-normal TSH concentrations (i.e. above roughly 2.5 mU/L). However, new studies published only shortly after release of the new guidelines could not show any beneficial effect of levothyroxine treatment for women with a TSH above 2.5 mU/L, but did find beneficial effects for women with a TSH above 4.0 mU/L [11–13]. However, larger studies are needed to confirm these findings and identify the true TSH concentration from which the outcome of clinical adverse outcomes is increased.

While much focus has gone into defining the upper limit for TSH, the definition of thyroid dysfunction is also dependent on the FT4 concentration. For example, in a hypothetical patient with a TSH of 5.5 mU/l, the FT4 concentration will decide whether there is overt hypothyroidism or subclinical hypothyroidism. The distinction between these clinical disease entities can have major consequences for the clinical work-up and approach. Although some studies have casted doubt about the validity of FT4 immunoassays during pregnancy, it is important to realize that the vast majority of patients present during early pregnancy during which the assay interference by thyroid hormone binding proteins is not relevant (only relevant during the third trimester). Moreover, lab-specific reference ranges for FT4 will still correctly identify women with true low or true high FT4 given that there is a high correlation between FT4 concentrations measured by immunoassays and after disequilibrium dialysis or with LCMS [1]. The alternative of increasing the non-pregnancy limits for total T4 by 150% does not seem viable given the gestational age specific changes and lack of association of total T4 with adverse outcomes [1, 14].

## Conclusions

In conclusion, any hospital or physician that is still using the 2.5 or 3.0 mU/l cut-off for TSH during pregnancy should re-evaluate their practice. When doing so, I strongly advise to start a study to define lab-specific references range for TSH and FT4. If there is absolutely no possibility to do so, a literature search to identify and adopt reference ranges from a similar lab would be the best alternative. Although it is highly likely that the use of the 2.5 and 3.0 mU/L cut-offs can lead to overtreatment, future studies are needed to identify if levothyroxine treatment in women with a TSH or FT4 outside of population-based reference ranges has beneficial effects.

## Abbreviations

ATA: American Thyroid Association
FT4: free thyroxine
TSH: thyroid stimulating hormone
